# LSM14A as a potential CSF biomarker for mild cognitive impairment and Alzheimer’s disease

**DOI:** 10.1002/alz.087990

**Published:** 2025-01-09

**Authors:** Sümeyra Ildız, Nazlıcan İlhan, Bedia Samancı, Pelin Sordu, Merve Alaylıoğlu, Hasmet Hanagasi, İbrahim Hakan Gurvit, Başar Bilgiç, Erdinc Dursun, Duygu Gezen‐Ak

**Affiliations:** ^1^ Institute of Neurological Sciences, Istanbul University‐Cerrahpasa, Istanbul Turkey; ^2^ Cerrahpaşa Faculty of Medicine, Istanbul University‐Cerrahpaşa, Istanbul Turkey; ^3^ Istanbul Faculty of Medicine, Istanbul University, Istanbul Turkey

## Abstract

**Background:**

mRNAs are required to progress cellular processes such as synaptic plasticity and memory formation. Processing bodies (P‐bodies) are storage units for mRNAs located at dendritic translation sites, so these mRNAs can participate in synaptic plasticity immediately when needed. P‐bodies consist of 3 main proteins: DDX6, 4E‐T, and LSM14A. It is thought that LSM14A is the part that binds to the mRNAs within the P‐bodies via its Lsm region, which is the region that recognizes and binds to mRNA. And so, LSM14A plays essential roles in mRNA translation, storage, and degradation. In neurodegenerative disorders, dendritic damage is seen in the early stages. It is unclear how much dendritic substance is released into the extracellular space after cellular damage. Some studies show the presence of exosomes and mRNA in the cerebrospinal fluid (CSF). The finding of mRNAs at dendrites with P‐bodies suggests that mRNAs could pass into the CSF via P‐bodies. In this study, we investigated the presence and the level of LSM14A in the CSF to determine its potential to be an early‐phase biomarker of Alzheimer's disease (AD).

**Method:**

In this study, the level of LSM14A protein was detected by ELISA in CSF of 21 patients with mild cognitive impairment (MCI), 40 patients with AD, and 18 cognitively unimpaired individuals. Raw data was analyzed by GraphPad InStat DTCG 3.06 with a one‐way ANOVA method followed by the Tukey‐Kramer posthoc test.

**Result:**

The level of LSM14A in the MCI group is significantly higher than in the control group, and the level of LSM14A positively correlated with patients' age. In AD patients, LSM14A levels increased in the first two years of the disease. LSM14A was negatively correlated with AD duration.

**Conclusion:**

According to our results, the level of LSM14A in the CSF is high in the MCIs. The disease duration of AD cases that were less than two years also showed elevated levels of LSM14A. Our preliminary results suggest that CSF LSM14A protein might be a CSF biomarker for the early stage of AD.

